# Optimizing aged care environments to promote resident functional mobility and reduce staff injury risk

**DOI:** 10.3389/fragi.2023.1157829

**Published:** 2023-04-06

**Authors:** Robyn Coman, Carlo Caponecchia

**Affiliations:** ^1^ Occupational Health and Safety (OHS) Academic Program, School of Health and Society, Faculty of Arts, Social Sciences and Humanities, University of Wollongong, Wollongong, NSW, Australia; ^2^ School of Aviation, Faculty of Science, University of New South Wales, Sydney, NSW, Australia

**Keywords:** manual handling, risk management, functional mobility, independence, well—being

## Abstract

**Introduction:** This study aimed to evaluate the suitability and usability of the Pro-Mobility patient/person handling assessment tool (ProMob) within residential aged care. Physiological changes associated with ageing influence an older person’s ability to perform functional mobility tasks such as transferring from furniture and walking. Strategies that improve capability and/or reduce the physical demands of the task have the potential to promote an older person’s mobility, independence and wellbeing. Environment-related strategies in Manual Handling of People (MHP), such as optimum seated heights, in part address this challenge, as they can promote resident functional mobility while also protecting staff from injury. The ProMob tool was developed to address this issue through systematic evaluation of these environmental factors.

**Methods:** The participants in this study were seven (7) residential aged care facilities (RACFs) operated by a not-for-profit aged care organization. A qualified assessor evaluated MHP risk management with the ProMob tool at each RACF through collection of data for a random sample of residents (*n* = 67) regarding their living environments and available mobility information. Data was transferred to an SPSS-22 statistical software database for analysis which involved descriptive statistics and cross tabulations.

**Results:** Application of the ProMob tool provided effective quantification of the nature and extent of environment-related MHP interventions that may influence resident mobility. Areas for improvement with MHP risk management were identified, with variation evident across RACF’s within the same organisation, which was not consistent with levels of care (e.g., lack of clear space to facilitate mobility). Low level care facilities were observed to have fewer adaptive environmental features that could potentially slow decline in independence.

**Discussion:** Features of the aged care environment can be used to facilitate the functional mobility of aged care residents, and simultaneously reduce injury risk for staff in MHP interactions. The ProMob tool can be used for auditing care facilities, planning re-development, and continual improvement in provision of care and management of staff injury risk exposure.

## 1 Introduction

Physiological changes associated with ageing influence an older person’s ability to perform mobility tasks such as transferring from furniture and walking (Mount et al., 2009). Strategies that improve capability (e.g., exercise) and/or reduce the physical demands of the task (e.g., assistive technology, assistance) have the potential to promote an older person’s mobility and wellbeing (Coman et al., 2018). At the same time, aged care workers who provide mobility assistance are exposed to high risk of musculoskeletal disorders (MSDs) associated with performance of these manual handling of people (MHP) tasks (Caponecchia et al., 2020; BLS, 2021). Risk management strategies implemented to address these risks have tended to focus on staff outcomes with limited consideration of outcomes for the resident such as promoting mobility (Fray, 2010; Taylor et al., 2011).

Reducing the exposure of staff to MSD risks is very important, given the high rate of MSDs internationally (Safework Australia, 2018; European Agency for Safety and Health at Work EU-OSHA, 2019; BLS, 2020). In Australia, MSDs continue to be the leading work-related problem, with “body stressing” (i.e., manual tasks) causing 37% of all serious workers compensation claims in 2020–2021 (Safework Australia, 2022).

As a task, MHP in aged care is different to other work contexts with hazardous manual tasks which result in MSDs, because the loads to be lifted or moved are older humans. These older humans have rights, expectations, feelings, abilities and limitations, and aged care facilities have a duty of care to them (Aged Care Quality and Safety Commission, 2021). Provision of care extends to clinical care, personal care (e.g., personal assistance with bathing/toileting) and ensuring the resident’s environment supports their independence. Finding ways to address these competing demands–providing appropriate levels of care and protecting staff from injury–is a key challenge in the aged care sector (Fray and Hignett, 2010; Taylor et al., 2011).

**FIGURE 1:**
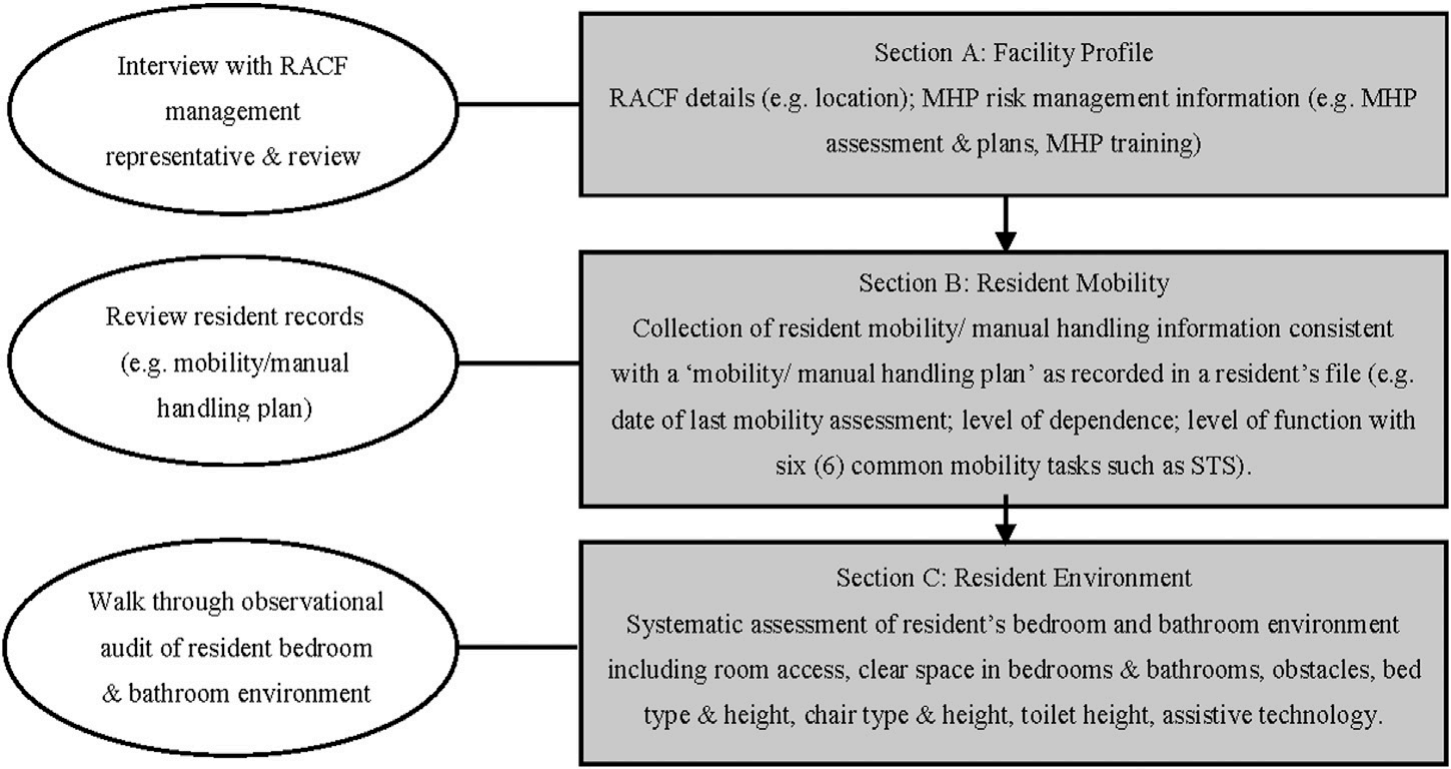
Outline of the sections of the ProMob Patient/Person Handling Assessment Tool and data used for the assessment (Coman and Caponecchia, 2015).

Environment-related strategies in MHP are a potential solution to this challenge, as they can promote resident mobility while also protecting staff from injury. These environment-related strategies can be relatively simple and cost effective such as optimum bed, chair and toilet heights to aid sit-to-stand (STS) transfers (Janssen et al., 2002; Mazza et al., 2004; Mathiyakom et al., 2005), and provision of assistive technology to assist the resident with transfers and movement within the bed (e.g., bed attachments to aid mobility). However, systematic use of these strategies for the dual benefits of reducing staff injury risk exposure and facilitating resident functional mobility (Figure 1), have not been fully realized (Coman and Caponecchia, 2015; Coman et al., 2018). The need to consider other MHP intervention outcomes, in addition to those associated with reducing MSD injury risk is emerging as a key issue within the MHP field (Pellatt, 2005; Nelson et al., 2008; Fray, 2010; Fray and Hignett, 2010; Fray and Hignett, 2010; Taylor et al., 2011; Taylor et al., 2014).

The Pro-mobility patient/person handling assessment tool (ProMob) was designed to extend existing MHP assessment methods by focusing on aspects of the resident’s environment which may contribute to promoting mobility and thus influence staff injury risk exposure. Content validity of this instrument was established through a modified Delphi study with a panel of MHP experts (Coman and Caponecchia, 2015).

Various methods have been applied within MHP risk management for identifying hazards associated with MHP tasks, assessing risks and evaluating interventions. These comprise a range of “patient handling assessment tools” that focus at different levels of the MHP interaction, including a focus on aspects of the individual patient (such as level of dependency); aspects of the work environment (e.g., furniture, architectural features); the individual nurse/care worker (such as skill and task performance); the organisation (e.g., safety management systems); or combinations of these elements (Fray, 2010; Coman et al., 2018).

Safe MHP task performance is typically informed by assessment of the patient’s current health status, care needs and the work area (e.g., ward, residential aged care bedroom), which should be recorded and communicated to staff through a manual handling plan (Queensland Health, 2021).

Some MHP assessment tools have been developed to evaluate MHP risks for care workers within a particular work unit/workplace, including the patient profile, patient handling equipment provision, staffing levels and environment related risks (e.g., clear space; fixed architectural features; floor surfaces) (Worksafe Victoria, 2007; ACC, 2012; Cantarella et al., 2020; Queensland Health, 2021). The potential impact of seated heights and patient handling equipment use on patient mobility has been considered (e.g., Battevi and Menoni, 2012; Cantarella et al., 2020), although evaluation of the nature and extent of environment-related strategies for facilitating mobility has been quite limited (Coman et al., 2018).

In contrast to existing tools, the ProMob tool focuses on indexing environment-related MHP interventions that have the potential to promote resident mobility and independence. It considers what information is available on mobility (which should be derived from an assessment by an appropriately qualified professional and should inform resident care) and then looks at the extent to which the environment supports mobility and facilitates that care. ProMob seeks to identify any inconsistencies that may exist between mobility assessment and resident environment. This study’s objectives were to evaluate the suitability and usability of the ProMob tool in residential aged care through a field trial undertaken at 7 RACF’s operated by the same provider.

## 2 Methods and materials

### 2.1 ProMob tool

The items assessed in the ProMob assessment tool and the methods by which they were assessed were based on extensive review of the MHP evidence (Coman et al., 2018). Based on the method for using the *MAPO Index* patient/person handling tool (Battevi and Menoni, 2012), ProMob assessors systematically evaluate a resident’s living environment within the context of MHP, after reviewing relevant records, and profiling the facility. As detailed in Figure 1, this involves an appropriately qualified assessor (e.g., a physiotherapist) collecting relevant information regarding the facility (Section A), resident mobility (Section B) and resident environment (Section C). Seated heights as a percentage of lower leg length are then determined using a calculator and recorded on the ProMob data collection sheet. Comparisons can then be made between mobility data and environmental information, for example, are the measured seat heights within a resident’s bedroom and bathroom environment (e.g., bed, chair, commode, toilet) within an optimum range for individual resident (i.e. 100%–120% of lower leg length/popliteal height). Quantified environment-related data can then be analysed in relation to resident mobility information to inform MHP intervention outcomes for the resident.

Of importance, collection of resident mobility information for Section B provides information consistent with a “mobility/manual handling plan”, but is not designed for assessment of resident mobility *per se*. ProMob is not an alternative to mobility assessment instruments, such as the *Physical Mobility Scale (PMS)* physiotherapy assessment tool (Nitz et al., 2006), but rather should be informed by them.

### 2.2 Recruitment process

The participants in this study were seven (7) RACFs operated by a not-for-profit aged care organization. Four facilities were located in major urban centers, two were in regional centers, and one was located in a small rural town. Size of the RACFs ranged from 40 beds to 149 beds. Two facilities provided high level care only (i.e., residents require complete assistance with most activities of daily living), three provided mixed care levels (high care and low care with “aging in place”), and two RACFs were low care (i.e., residents require accommodation, meals, laundry, room cleaning, some assistance with personal care) only. Assessment of MHP risk management with the ProMob tool at each RACF was undertaken through collection of data for a random sample of ten (10) residents who were classified as either “independent” (no assistance required from care staff) or “able to assist” (can perform task but requires some assistance from care staff) with mobility. Changes in health status during the data collection process, resulted in inclusion of information for 67 residents from the RACFs assessed.

### 2.3 Procedure

During a site visit to each RACF, a ProMob assessment was undertaken by the organization’s Physiotherapy Coordinator (i.e., the “assessor”). The assessor was provided with 2 - 3 hours of training in use of the tool; extensive guidance material and relevant resources; and practical assistance with collection of data. The assessor was responsible for organizing all site visits and collecting all data (Sections A, B and C).

For Section B of the tool, basic mobility data was collected by the assessor from the resident’s records and was consistent with what is typically required for a manual handling/mobility plan (i.e., plan for how resident is assisted). This included a rating of overall mobility status using Worksafe Victoria criteria (i.e., independent, able to assist, dependent), and rating of performance with six common mobility tasks: 1) sitting up/lying down (in bed); 2) repositioning (turning) in bed; 3) transferring from lying to sitting on side of bed; 4) sit-to-stand (STS) transfer from bed; 5) STS transfer from chair; 6) walking (Worksafe Victoria, 2007; ACC, 2012; Queensland Health, 2021). Individual mobility tasks were rated on level of assistance from staff (independent; assist of 1 staff; assist of 2 staff) and use of equipment (e.g., assistive technology).

The “Stick-to-stand” measurement instrument (Compact Business Systems, 2020) was used to assess individual seated height requirements for each resident, through measurement of lower leg length (LLL) when sitting (i.e., seated knee height).

The selected residents’ bedroom and bathroom living environments were evaluated during a walkthrough observational assessment. Spatial dimensions and furniture heights were measured using appropriate measurement equipment (5 m metal tape and a 1,200 mm spirit level with rule).

Following completion of all assessments the assessor provided feedback to the researcher through a structured questionnaire regarding the usefulness of the instrument and any suggested changes.

### 2.4 Statistical analysis

ProMob assessment data were recorded in separate hard copy data collection booklets for each section of the tool for the entire RACF. Data was transferred to an *SPSS-22* statistical software database for analysis which involved descriptive statistics and cross tabulations.

### 2.5 Ethical considerations

The study was approved by the appropriate ethics committee.

## 3 Results

### 3.1 Facility profile (Section A)

At the seven RACFs mobility/manual handling assessments were undertaken every 6 months for all residents and/or when changes in mobility occurred, and were the principal responsibility of the Physiotherapists employed at each facility. Mobility status was recorded in “care plans” and “mobility and dexterity forms”. Care plans did not include directives for care staff in relation to bed and seating heights. Resident manual handling plans were stored in residents’ rooms at most facilities. Physiotherapists and/or senior nursing staff provided advice to care staff regarding manual handling. Mandatory annual manual handling training for staff was risk management based (Safework Australia, 2018) and included body mechanics, MHP techniques and use of MH equipment.

### 3.2 Key findings from analysis of associations between resident mobility (section B) and environmental factors (section C)

A range of mobility levels, as assessed by the Worksafe Victoria mobility criteria were observed across high and low care facilities (see Table 1).

**TABLE 1:** Summary of overall dependency/mobility status of randomly selected residents at all RACFs classified as independent or able to assist.

Level of care	RACF ID	Number of residents	Mobility level: WorkSafe Vic. (% residents)	
			Independent	Able-to-assist
High care only	F1	9	22.2	77.8
	F2	9	22.2	77.8
High care/low care RACF (with “ageing-in-place”)	F3	10	100	
	F4	10	60	40
	F5	10	60	40
Low care only	F6	10	100	
	F7	9	100	

Variation in practice was evident across the RACFs in relation to environmental factors such as furniture heights and assistive technology (AT) use. As detailed in Table 2, provision of electric height adjustable beds which can reduce staff injury risk exposure and aid resident mobility, was common in the high care (F1 and F2) and mixed care (F3, F4 and F5) facilities. By contrast in the two low care facilities (F6 and F7), non-adjustable domestic furniture was almost exclusively observed. Use of bed AT to aid mobility with on-bed tasks and STS transfers to/from the bed, was limited at most RACFs, with overhead trapeze AT principally observed (Table 3). The assessor reported that bedstick/poles were rarely used owing to entrapment concerns within the aged care sector following recent reports of bedstick/pole related fatalities (Australian Coroners Court, 2010). Only two of the randomly selected residents at one low care RACF (F7) used a bedstick/pole. However, as can be seen in Table 2, bed side-rails, which have also been associated with patient safety issues such as entrapment and falls (Hignett and Masud, 2006; Capezuti et al., 2008; Hignett, 2010), were fitted to a majority of beds at high care and high care/low care facilities.

**TABLE 2:** Summary of the relationship between level of performance with sitting up/lying down (Task 1) and key environmental factors.

Task: Sitting up/lying down (task 1)			RACF (% of residents)						
			High care RACF		High care/low care RACF			Low care RACF	
Level of performance	Environmental factors		F1	F2	F3	F4	F5	F6	F7
Independent	Bed type	Non-height adj			50			100	88.9
		Height adj. electric	100	100	50	100	100		11.1
	Bed side rails	One				14.3			
		Both	100	80	20	71.4	87.5		
		Nil		20	80	14.3	12.5	100	100
	Clear space	650 mm at both sides	20		20	71.4	37.5	30	
	Obstacles		20	60	70	42.9	12.5	70	22.2
	Equipment use	Trapeze			30				
		Bed stick/pole							11.1
Able to assist (1–2 staff)	Bed type	Non-height adj							
		Height adj. electric	100	100		100	100		
	Bed side rails	One							
		Both	100	75		66.7	50		
		Nil		25		33.3	50		
	Clear space	650 mm at both sides	25			66.7			
	Obstacles			100			100		
	Equipment use	Trapeze		33.3					
		Bed stick/pole							

**TABLE 3:** Summary of the relationship between level of performance with transferring from lying to sitting on side of bed (Task 2) and key environmental factors.

Task: Transferring from lying to sitting on side of bed (task 2)			RACF (% of residents)						
			High care RACF		High care/low care RACF			Low care RACF	
Level of performance	Environmental factors		F1	F2	F3	F4	F5	F6	F7
Independent	Equipment use*	Trapeze			40				11.1
		Bed stick/pole							11.1
Able to assist (1–2 staff)	Equipment use*	Trapeze		16.7		33.3	25		
		Bed stick/pole							

Clear space around beds was frequently observed to be non-optimum due to room dimensions, fittings and fixtures, and/or heavy furniture. Obstacles within the bed space (i.e., fixed or immovable objects) were identified to varying extents at all facilities. Clear space on both sides of the bed of at least 650 mm for staff access (WorkSafe Victoria, 2009), was not available for residents assessed who required assistance with Task 1 (sitting up/lying down) at F2 and F5. Clear space of 900 mm on one side of the bed (Worksafe Victoria, 2007) was provided for over half the residents able-to-assist (57.1%) at one facility (F1). However, at another high care facility (F2) all bed spaces provided less than 900 mm clear space on at least one side due to the impact of shared bedrooms, dividing cubicle curtains and positioning of furniture.

Analysis of STS transfer ability in mobility assessments identified environment-related factors that may influence resident MHP intervention outcomes, such as bedroom chair type which varied widely. Most of the seating provided across all RACFs was fixed height seating. Excluding electrically operated chairs, use of chairs of an optimum height range by residents “able to assist” with STS transfer ranged from 33.3% at F4 to 100% at F5. Backward sloping seats that may increase STS task demands were in place by a large majority of residents “able to assist” with STS (Table 4).

**TABLE 4:** Summary of the relationship between level of performance with STS transfer from chair (Task 3) and key environmental factors.

Task: STS transfer from chair (task 3)			RACF (% of residents)						
			High care RACF		High care/low care RACF			Low care RACF	
Level of performance	Environmental factors		F1	F2	F3	F4	F5	F6	F7
Independent	Chair height as a % of LLL^a^	Less than 100% LLL			14.3			28.6	50
		100%–120% LLL	50	66.7	71.4	57.1	100	57.1	50
		121%–140% LLL	50	33.3	14.4	42.9		14.3	
	Chair type^b^	Height adj	33.3				20	16.7	
		Electric			37.5		20	16.7	83.3
		Fixed height		100	50	83.3	40	66.7	16.7
		Other	33.3		12.5	16.7	20		
		No chair	33.3						
	Chair foot clearance	Adequate	100	100	57.1	83.3	75	20	25
	Chair seat slope	Horizontal	100	100	57.1	71.4	50	50	50
		Backward			42.9	28.6	50	50	50
	Castors^c^	No brakes			11.1			28.6	
	Equipment use^d^	Walkbelt	N/A	N/A	N/A	N/A	N/A	N/A	N/A
Able to assist	Chair height as a % of LLL^a^	Less than 100% LLL		20					
		100%–120% LLL	66.7	60		33.3	100		
		121%–140% LLL	33.3	20		66.7			
	Chair type^b^	Height adj	16.7						
		Electric	16.7				66.7		
		Fixed height		83.3		100	33.3		
		Other	33.3	16.7					
		No chair	33.3						
	Chair foot clearance	Adequate	100	100		66.7	100		
	Chair seat slope	Horizontal	66.7	40		50			
		Backward	33.3	60		50	100		
	Castors^c^	No brakes		0		33.3			
	Equipment use^d^	Walkbelt		33.3					0

^a^ Excludes electrically operated chairs. No chairs were greater than 140% LLL.

^b^ All bedroom chairs had armrests.

^c^ No castors with brakes observed.

^d^ Use of walkbelt to assist with transfer was the only equipment reported.

Analysis of STS transfer performance and toilet heights found that residents at a majority of RACFs used toilets of an optimum height range. However at one low care facility (F7) two-thirds of residents used toilets that were lower than 100% of LLL, all of whom were independent in STS transference. Lower toilet heights (less than 100% of LLL) were also used by a minority of residents “able to assist” with STS transfer at F1 (33.3% residents) and F2 (16.7% of residents). Use of toilet AT other than grab rails to aid STS transference was limited to a small percentage of residents, and use was inconsistent across facilities. Height adjustable toilet surrounds with seat were the main type of AT observed and were principally used by residents who were independent in STS transference.

### 3.3 Assessor feedback

Following collection of data with the ProMob Tool, the assessor provided feedback to the researcher in a semi-structured interview. The ProMob method was considered to be simple yet effective, and provided appropriate information for investigation of environment-related MHP interventions that may influence patient/resident mobility outcomes. Training in use of the tool, particularly discussion of research evidence that supports inclusion of particular items, was reported to be of value in understanding of the importance of this aspect of MHP risk management. The assessor suggested that background information could include “a summary sheet of common aids, including pros/cons” and further information regarding “common mobility issues”.

Regarding suitability for application in aged care, the assessor rated the ProMob Tool as 8/10 on a scale from 1 (not suitable at all) to 10 (very suitable). Of importance, the assessor commented that the tool could be used “… for identifying issues and promoting discussion on appropriateness of aids and potential for relocation of furniture in order to improve space issues.”

Assessment of resident environments with the ProMob tool was considered to provide a detailed evaluation of the field trial sample, but could be time consuming if extended to more residents. They suggested that efficiency of environmental data collection could be improved through use of hand held devices (e.g., iPad), with “drop down” menu options and integrated camera functions for recording particular aspects. It was also suggested that the components of the tool could be used for assessment of specific aspects such as “…Chair evaluation ProMob Tool”; “Bed/bed surrounds ProMob tool”. The assessor considered potential applications of the ProMob Tool in provision of care could include hazard identification, implementation and evaluation of MHP risk control interventions, and training needs analyses.

## 4 Conclusion

The ProMob Tool was developed to index environment-related MHP interventions that may influence mobility, as existing instruments provided limited coverage of this aspect of MHP risk management. This investigation found aspects of MHP risk management could be improved to promote resident mobility and reduce care staff exposure to MHP related injuries. In addition, variation was evident within and between facilities in a manner which was not consistent with levels of care (e.g., chairs with backward sloping seats, and limited adjustable height seating in high care facilities). Clear space for performance of MHP tasks and for resident mobility was frequently compromised, provision of optimum seated heights to aid STS transfers varied widely and provision of AT to aid mobility was limited.

Feedback from the assessor highlighted the suitability of the ProMob tool for identifying these MHP related environmental issues and promoting discussion on how furniture could be relocated to improve clear space for movement of resident and staff.

Several of the results reflect common issues in MHP research and practice. Bed height, and the ease with which bed height can be adjusted is recognized as one of the major factors that can facilitate transfers from beds (Capezuti et al., 2008; Oxley et al., 2010).

Use of electrically operated beds was observed for all residents assessed in high care RACFs, most residents in mixed care RACFs, yet very few residents in low care RACFs where domestic furniture was common. Residents randomly selected at the low care RACFs (F6 and F7) were all independent with mobility task performance. However, further investigation of bed provision in these RACFs is warranted with consideration of the comments from care staff at these sites, who identified non-height adjustable domestic beds as a major MHP issue for staff and residents.

Bedstick/pole bed AT to aid mobility continues to be a contentious issue in RACFs following several bedstick/pole related injuries and fatalities. Limited use was evident in this study with bedstick/poles only identified on two beds at F7. Use of overhead trapeze bed AT by residents “able to assist” with repositioning in bed (Task 2), was observed with a third of residents assessed at F2 and half of residents assessed at F4 and F5. This type of AT provides limited benefit with transferring from supine to sitting (Task 3) and/or STS transfers from the bed (Task 4). This was evident with findings of limited use (e.g., no residents “able to assist” with Task 4 used the trapeze). Study findings and limited research evidence regarding the importance of bed AT to aid transfers to/from the bed support the need for further investigation of bedstick/pole assisted transfers (Alexander et al., 2000; Morse et al., 2015).

Seated heights are a key determinant of STS transfer ability for the older person (Janssen et al., 2002). Height adjustable seating enables seat heights to be adjusted as the resident’s mobility status changes. It is of interest to note that use of height adjustable seating was limited, if not absent, at high care facilities (F1 and F2) where care needs of residents are higher. Wide variation between RACFs was also evident with provision of optimum seat heights for residents who required assistance with Task 5: STS transfer from chair (33.3%–100%).

Transfers on/off toilets are frequently performed MHP tasks that expose care workers to high risk of injury. Evaluation of toilet environments in this study demonstrated clear space around toilets was adequate at most RACFs for residents who required assistance from care staff. Both low care RACFs provided limited clear space owing to structural features consistent with older facilities. Limited use of this evidence-based and cost effective strategy warrants further investigation by the organisation. Ensuring all seated heights are of an optimal height for the individual resident can significantly reduce task demands and need for assistance.

### 4.1 Use of ProMob analysis by RACFs

The data generated by the ProMob assessment tool can be used by the organization operating the RACFs, and by local managers, to identify opportunities for improvement. This includes addressing a lack of consistency in environment-related issues for residents at particular care levels; provision of clear space, provision of AT and use of bedrails, and assessment and provision of furniture and infrastructure with appropriate seated heights to facilitate mobility.

Comparison of environment-related MHP intervention strategies between RACFs operated by the same organization demonstrated another potential application of the ProMob tool. Inconsistencies within and between RACFs operated by the same provider could be explained by differences in professional opinion or practice among senior nursing staff and/or safety culture within specific RACFs. Similarly, comparing the features of high and low care facilities could be useful for organizations. While it was observed, as expected, that there were some differences between low and high care (e.g., more height adjustable chairs in high care), the care needs of residents can quickly change, and appropriate environmental features may help residents stay in low care for longer. With this in mind, the provision of optimized environmental features such as adjustable/optimum bed, seat and toilet heights for residents rated as independent and those in low care facilities, could be a proactive strategy to slow the decline in independence. In other words, use of the ProMob tool to identify inconsistencies across types of care facilities can improve overall care. While high care facilities should be most adapted to mobility needs, there is also a strong argument for facilitating mobility through care environment features in all types of care.

As was highlighted by the assessor, systematic assessment with the ProMob across the organization would inform continuous improvement with MHP risk management, assist with identification of staff training needs, and may provide information regarding potential patient quality of care outcomes. Importantly, it was noted in Section A of the ProMob evaluation that resident care plans did not include directives for care staff in relation to bed and seating heights.

Evidence from assessment with the tool, and user feedback from the assessor, supported use of the ProMob tool by appropriately qualified professionals responsible for mobility/manual handling assessments within RACFs. The assessor commented during site visits that the ProMob inspections provided a unique insight into the care environment at the different facilities, which had not been possible previously with the assessment instruments and systems used within their professional role as Physiotherapy Coordinator. ProMob thus enabled a consistent assessment of practice across the organization, in a manner that had previously relied on the implementation of manual handling plans for individual residents. While such plans are standard practice in the industry, they do not trigger staff to identify environmental improvements which if systemically applied could facilitate mobility and reduce the risk of injury.

There are a number of other potential practical uses for the ProMob tool including:

• Identification of environment-related MHP issues that may influence patient mobility outcomes, to inform risk control interventions.
• Evaluation of the effectiveness of existing environment-related MHP interventions.
• Identification of staff training needs in relation to environment-related MHP strategies that may influence staff and resident safety. For example, recommendations for optimum seating heights (Weiner et al., 1993; Alexander et al., 2000), and recommended amounts of clear space (Hignett, 2005).
• Identification of potential quality of care outcomes associated with MHP interventions.
• Audits of environmental features of RACFs when planning refurbishment of the built environment.

### 4.2 Limitations and future research

Several limitations are evident in the present study. Only 7 RACFs were included in the field test, and 67 resident environments and records were analyzed. While inclusion of additional RACFs may have shown greater variation in practice, the main aim of the study was to evaluate use of the tool. Accordingly, using the tool in a range of facilities controlled by one organization provided a context that minimized organizational variation, while still testing the features of tool across diverse facilities. Further research could aim to explore practice in relation to environment-related features for MHP across organizations or within health jurisdictions, using the tool to collect relevant data. Similarly, only one assessor was used to collect data with the ProMob tool. Most organizations using the tool would likely only have one assessor, or they may have several staff agreeing on ratings together. Models for the implementation of ProMob, including assessment of inter-rater reliability could be explored in further research, which could provide certainty on comparability of results should the tool be used by multiple assessors across facilities within a single organization. The assessor also provided several suggestions for improvements to the tool, including digitization and customization that may need to be assessed for usability in future. Evaluating the implementation of changes based on ProMob analysis of an RACF would also be important for further medium and long-term research, and could include potential impacts on injury rates, staff workload, and resident satisfaction and care outcomes.

In conclusion, features of the aged care environment can be used to facilitate the mobility of aged care residents, and simultaneously reduce injury risk for staff in MHP interactions. While existing MHP tools consider a range of perspectives, environment-related strategies such as seated heights and clear space around furniture have not been systematically assessed. Use of the ProMob tool in 7 RACFS found divergent practices in clear space, furniture type and features, and assistive technology. The tool was rated highly by the assessor as being useful for the evaluation of environment-related MHP interventions that may influence resident mobility outcomes, which can assist RACFs in managing risks and improving care. Further research on MHP practices in aged care can be facilitated by the ProMob tool, in addition to being used for auditing current practices.

## Data Availability

The original contributions presented in the study are included in the article/supplementary material, further inquiries can be directed to the corresponding author.
